# Association of air pollution with outpatient visits for respiratory diseases of children in an ex-heavily polluted Northwestern city, China

**DOI:** 10.1186/s12889-020-08933-w

**Published:** 2020-06-02

**Authors:** Yueling Ma, Li Yue, Jiangtao Liu, Xiaotao He, Lanyu Li, Jingping Niu, Bin Luo

**Affiliations:** 1 Institute of Occupational Health and Environmental Health, School of Public Health, Lanzhou University, Lanzhou, Gansu 730000 People’s Republic of China; 2 Gansu Provincial Maternity and Child Health Care Hospital, Lanzhou, Gansu 730000 People’s Republic of China; 3 Shanghai Typhoon Institute, China Meteorological Administration, Shanghai, 200030 China; 4 Shanghai Key Laboratory of Meteorology and Health, Shanghai Meteorological Bureau, Shanghai, 200030 China

**Keywords:** Air pollution, Respiratory diseases, Children, Outpatient visit, Time-series study

## Abstract

**Background:**

A great number of studies have confirmed that children are a particularly vulnerable population to air pollution.

**Methods:**

In the present study, 332,337 outpatient visits of 15 hospitals for respiratory diseases among children (0–13 years), as well as the simultaneous meteorological and air pollution data, were obtained from 2014 to 2016 in Lanzhou, China. The generalized additive model was used to examine the effects of air pollutants on children’s respiratory outpatient visits, including the stratified analysis of age, gender and season.

**Results:**

We found that PM_2.5_, NO_2_ and SO_2_ were significantly associated with the increased total respiratory outpatient visits. The increments of total respiratory outpatient visits were the highest in lag 05 for NO_2_ and SO_2_, a 10 μg/m^3^ increase in NO_2_ and SO_2_ was associated with a 2.50% (95% CI: 1.54, 3.48%) and 3.50% (95% CI: 1.51, 5.53%) increase in total respiratory outpatient visits, respectively. Those associations remained stable in two-pollutant models. Through stratification analysis, all air pollutants other than PM_10_ were significantly positive associated with the outpatients of bronchitis and upper respiratory tract infection. Besides, both NO_2_ and SO_2_ were positively related to the pneumonia outpatient visits. PM_2.5_ and SO_2_ were significantly related to the outpatient visits of other respiratory diseases, while only NO_2_ was positively associated with the asthma outpatients. We found these associations were stronger in girls than in boys, particularly in younger (0–3 years) children. Interestingly, season stratification analysis indicated that these associations were stronger in the cold season than in the transition or the hot season for PM_10_, PM_2.5_ and SO_2_.

**Conclusions:**

Our results indicate that the air pollution exposure may account for the increased risk of outpatient visits for respiratory diseases among children in Lanzhou, particularly for younger children and in the cold season.

## Background

Air pollution is one of the greatest environmental risks to public health. The World Health Organization (WHO) report showed that outdoor air pollution was responsible for 4.2 million deaths worldwide in 2016 [1]. A growing body of literature has investigated the association between air pollution and respiratory tract, which is the main organ affected by air pollution. For instance, a panel study from Korea suggested that air pollution may cause respiratory symptoms [2]. In addition, a considerable amount of papers have focused on the associations between air pollution and respiratory diseases/mortality in Europe [3, 4], the United States [5, 6], and some Asian countries [7, 8]. In Taiwan, two main air pollutants (NO and NO_2_) were positively associated with respiratory diseases, followed by PM_10_, PM_2.5_, O_3_, CO and SO_2_ [9]. A study with urban Chinese population found that per 10 μg/m^3^ increase in PM_2.5_ and PM_10_ concentration on the current day of exposure was associated with 0.36 and 0.33% increase in respiratory system disease, respectively [10]. In Hangzhou, outpatient visits of adults with respiratory disease increased by 0.67, 3.50 and 2.10% with per 10 μg/m^3^ increase in PM_2.5_, SO_2_ and NO_2_, respectively, however, children outpatient visits increased by 1.47, 5.70 and 4.04%, respectively, which indicated that children were more susceptible to air pollutants [11]. Besides, the results for a study in Taiwan showed significant relationships between NO_2_, PM_10_ and asthma outpatients, especially for children [12]. Therefore, air pollution may affect the respiratory outpatient visits.

Children have relatively immature lungs and immune system, and inhale a larger volume of air per body weight [13], so they are more susceptible to the adverse respiratory effects of air pollution. Exposure to air pollution at early stage may affect children’s normal growth and lung development [14, 15]. The increased prevalence of young children’s respiratory diseases was also related to air pollution exposure time and dose in Jinan [16]. Particularly, air pollution was positively related to the pneumonia among children [17, 18]. Besides, better air quality has been approved to reduce respiratory symptoms among children [19]. However, research about comprehensive comparison of respiratory health changes in children from different subgroups is still limited, especially in cities that suffer from heavy air pollution.

Air pollution is a global problem. About 91% of the world population was estimated to breathe polluted air which exceeded the WHO air quality guideline levels in 2016 [20]. Lanzhou, an industrial city, located in a typical valley basin, is particularly well known as a dry city with scarce rainfall, high evaporation and low wind speeds [21]. Moreover, it is also frequently affected by dust storms due to its location closed to the arid and semi-arid region of Northwest China [22]. These factors combine to make Lanzhou one of the most traditional seriously air-polluted cities in China. Although, a study with very limited data has reported the effect of PM_2.5_ over respiratory disease in Lanzhou, but didn’t focus on the children [21]. Normally, children are often divided into young children period (0–3 years age), preschool period (4–6 years age) and school period (7–13 years age), displaying growing level of immunity, who may show different effects when exposed to air pollution [23]. Therefore, we aim to assess the effects of air pollutants on children’s outpatient visits for respiratory diseases from different subgroups with the data of 15 hospitals in a poor area of China-Lanzhou city.

## Methods

### Study area and data collection

Being the capital city of Gansu province, Lanzhou is located in the north-west of China with a population of over 3.7 million in 2017 [24]. Lanzhou is one of the most air-polluted cities in China, because it is heavily industrialized and owns a valley style terrain, and has a typical semi-arid continental climate with scarce precipitation [21, 25]. Even though the authorities have taken significant measures to improve the air quality in Lanzhou, the level of air pollutants concentration (The average annual PM_2.5_, PM_10_, SO_2_ and NO_2_ concentrations during 2007–2016 in Lanzhou were 61.23 μg/m^3^, 136.14 μg/m^3^, 42.93 μg/m^3^ and 45.37 μg/m^3^, respectively.) [21] exceeded the national level II (The average annual standards for PM_2.5_ is 35 μg/m^3^, PM_10_ is 70 μg/m^3^, SO_2_ is 60 μg/m^3^, and NO_2_ is 40 μg/m^3^.).

**Fig. 1:**
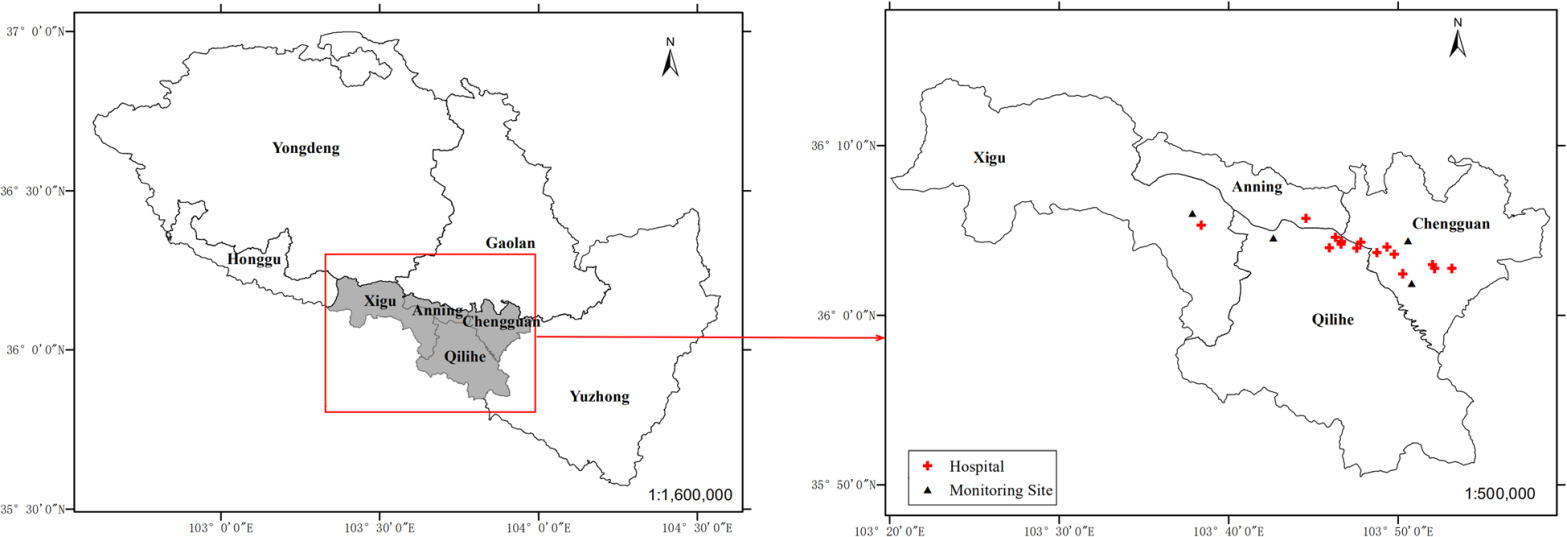
Spatial distribution of air quality monitoring stations, studied hospitals, and four central urban districts in Lanzhou, China. Source: The map was created by the authors with ArcGIS 10.2.2 software (ESRI, Redlands, California, USA). ArcGIS is the intellectual property of ESRI and is used by license in here

The daily number of outpatients for respiratory diseases between 2014 and 2016 were obtained from the 15 hospitals of the four central urban districts of Lanzhou (Chengguan, Qilihe, Xigu and Anning) (Fig. 1), which was confirmed and permitted by the Lanzhou center for disease control and prevention. This study protocol was approved by the ethics committee of Lanzhou University (Project identification code: IRB190612–1). We screened the outpatient visit data using the 10th Revision of the International Classification of Diseases (ICD-10) Code of respiratory diseases (J00-J99). We excluded the patients who were not living in the four central urban districts of Lanzhou and those children aged ≥14 years. Finally, all outpatient data were classified into four specific diseases [pneumonia, J12-J18; asthma, J45-J46; bronchitis and upper respiratory tract infection (J00-J06, J20-J21, J30-J39, J40-J42); and other respiratory diseases (J22, J43-J44, J47, J60-J99)].

The simultaneous daily meteorological variables and air pollutants data were obtained from open access website of Lanzhou Meteorological administration and Lanzhou air quality monitoring stations (including Institute of Biology, Railway design institute, Hospital of Staff and LanLian Hotel) (Fig. 1), respectively. The air quality monitoring stations were in four central urban districts of Lanzhou. Meteorological variables included daily average temperature and relative humidity, and air pollutants data included particulate matter with aerodynamic diameter ≤ 10 μm (PM_10_), particulate matter with aerodynamic diameter ≤ 2.5 μm (PM_2.5_), nitrogen dioxide (NO_2_) and sulfur dioxide (SO_2_).

### Statistical methods

The descriptive analysis was performed for all data. The Quasi-Poisson regression with generalized additive model (GAM) was used to examine the associations between air pollutants (PM_10_, PM_2.5_, NO_2_ and SO_2_) and the daily children’s outpatient visits with respiratory diseases. The Quasi-Poisson distribution was applied to overcome the overdispersion of outpatient visits data. Generalized additive model allows for highly flexible fitting as the outcome is supposed to be dependent on a sum of the smoothed and linear functions of the predictor variables [26]. Based on the previous studies, the penalized smoothing spline function was used to adjust for long-term time trends, day-of-week, holiday and meteorological factors [27, 28]. The basis GAM equation is:

$$ {\displaystyle \begin{array}{c}\mathrm{logE}\left({Y}_t\right)=\upalpha +\upbeta {X}_t+s\left( Time,k= df+1\right)+s\left({Temperature}_l,k= df+1\right)\\ {}+s\left({Humidity}_l,k= df+1\right)+ DOW+ Holiday\end{array}} $$

Where t is the day of the observation; E (Y_t_) is the expected number of daily outpatient visits for respiratory diseases on day t; *α* is the intercept; *β* is the regression coefficient; X_t_ is the daily concentration of air pollutant on day t; s() denotes the smoother based on the penalized smoothing spline; The same lag structures as pollutants for temperature and relative humidity are adjusted, and Temperature_l_ and Humidity_l_ are the six-day moving average (lag 05) of temperature and relative humidity, respectively [27, 29]. Based on Akaike’s information criterion (AIC), the 7 degrees of freedom (*df*) per year is used for long-term time trends and 3 df for Temperature_l_ and Humidity_l_; DOW is a categorical variable indicating the date of the week; and holiday is a binary variable for national holiday in China.

After constructing the basic model, single-pollutant models were used to examine the lagged effects, i.e., single day lag (from lag 0 to lag 5) and multiple-day average lag (from lag 01 to lag 05). A spline function of GAM was applied to plot the exposure and response correlation curves between air pollution and outpatient visits for respiratory diseases. Moreover, two-pollutant models were set to evaluate the robustness of our results after adjusting for the other pollutants. In stratification analysis, all of these outpatients were classified into different sex (boys and girls) and age (0–3 years, 4–6 years and 7–13 years), and season [cold season (November to March), hot season (June to August) and transition season (April, May, September and October)] [23, 30]. According to the AIC and previous studies [23, 31], the *df* of time was 3, 2, 3 per year for the cold, hot and transition season, respectively. We also conducted a sensitivity analysis by changing the *df* from 5 to 9 per year for calendar time and from 3 to 8 for temperature and relative humidity.

All the statistical analyses were two-sided, and at a 5% level of significance. All analyses were conducted using R software (version 3.5.2) with the GAM fitted by the “mgcv” package (version 1.8–26). The effect estimates were denoted as the percentage changes and their 95% confidence intervals (CIs) in daily children’s outpatient visits for respiratory diseases associated with per 10 μg/m^3^ increase in air pollutant concentrations. The ArcGIS 10.2.2 software (ESRI, Redlands, California, USA) and GraphPad Prism 7.00 software were used to plot the Figures.

## Results

### Descriptive of air pollutants, meteorological variables and respiratory diseases outpatient data

There were 332,337 respiratory diseases outpatient visits for children during January 1st, 2014 through December 31st, 2016 in 15 major hospitals of Lanzhou. The mean concentrations of PM_2.5_, PM_10_, SO_2_ and NO_2_ were 54.52 μg/m^3^, 123.35 μg/m^3^, 22.97 μg/m^3^ and 51.80 μg/m^3^ during 2014–2016, respectively. In addition, the median of temperature and relative humidity were 12.9 °C and 50%, respectively (Table 1). On average, there were approximately 303 respiratory diseases outpatient visits per day in our study areas, and the bronchitis and upper respiratory tract infection, boys, children aged 4–6 and 7–13 years, and cold season had higher visits than other groups (Table 2).

**Table 1:** Descriptive statistics on daily air pollutants and meteorological parameters

Variables	Mean (SD)	P_25_	P_50_	P_75_	Range
Air pollutants (μg/m^3^)					
PM_2.5_	54.52 (28.42)	36.01	46.60	66.52	7.15–299.06
PM_10_	123.35 (75.69)	82.39	108.36	144.88	19.72–1207.13
SO_2_	22.97 (15.06)	11.47	19.06	31.12	3.47–130.59
NO_2_	51.80 (21.26)	36.07	49.34	63.26	12.25–138.15
Meteorological parameters					
Temperature (°C)	11.40 (9.75)	2.58	12.90	19.70	−12.40 – 29.80
Relative humidity (%)	50.58 (14.57)	39.00	50.00	61.00	16.00–88.00

*SD* standard deviance, *P*_*25*_ 25th percentile, *P*_*50*_ 50th percentile, *P*_*75*_ 75th percentile, *PM*_*2.5*_ particulate matter with aerodynamic diameter ≤ 2.5 μm, *PM*_*10*_ particulate matter with aerodynamic diameter ≤ 10 μm, *NO*_*2*_ nitrogen dioxide, *SO*_*2*_ sulfur dioxide

**Table 2:** Descriptive statistics on daily outpatient visits in Lanzhou, China, during 2014–2016

Variables	Mean ± SD	Min	P_25_	P_50_	P_75_	Max
Outpatient visits (cases/per day)						
Total	303.23 ± 97.97	95.00	234.00	292.00	357.00	732.00
Pneumonia	21.48 ± 15.77	0.00	10.00	17.00	28.00	95.00
Asthma	8.49 ± 5.43	0.00	5.00	7.00	11.00	36.00
Bronchitis and upper respiratory tract infection	265.28 ± 83.04	85.00	209.00	260.00	312.00	668.00
Other respiratory diseases	7.98 ± 5.72	0.00	4.00	7.00	11.00	37.00
Sex						
Boys	174.43 ± 55.32	42.00	135.00	170.50	205.00	418.00
Girls	128.79 ± 44.38	42.00	97.00	124.00	154.00	314.00
Age (years)						
0–3	28.45 ± 42.42	0.00	0.00	6.00	49.00	229.00
4–6	136.33 ± 42.76	43.00	103.00	134.00	167.00	262.00
7–13	138.45 ± 58.27	24.00	95.00	133.00	173.00	423.00
Season						
Cold (Nov to Mar)	351.60 ± 115.54	95.00	266.25	355.00	422.75	732.00
Transition (Apr, May, Sep and Oct)	285.93 ± 50.26	163.00	252.00	283.00	322.00	444.00
Hot (Jun to Aug)	246.60 ± 73.46	114.00	190.00	233.50	297.25	442.00

**Fig. 2:**
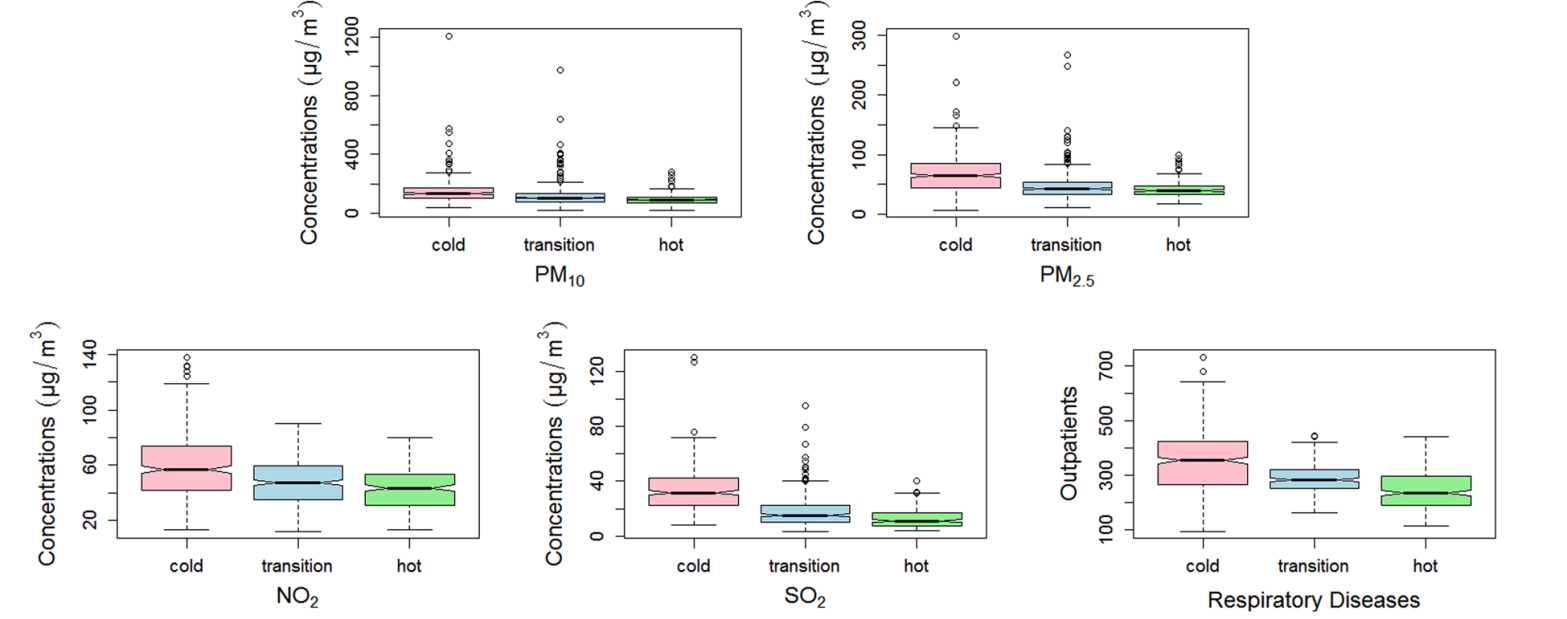
Box plots of air pollutants and total outpatients with respiratory diseases in the cold, transition and hot season. Boxes indicate the interquartile range (25th percentile-75th percentile); lines within boxes indicate medians; whiskers below boxes represent minimum values; whiskers and dots above boxes indicate maximum values. PM_2.5_, particulate matter with aerodynamic diameter ≤ 2.5 μm; PM_10_, particulate matter with aerodynamic diameter ≤ 10 μm; NO_2_, nitrogen dioxide; SO_2_, sulfur dioxide

Figure 2 showed that daily air pollutant concentrations were higher in the cold season than in the hot season, such as, the interquartile range of PM_10_, PM_2.5_, NO_2_ and SO_2_ concentrations in the cold season were 70.20 μg/m^3^, 41.00 μg/m^3^, 32.20 μg/m^3^ and 20.00 μg/m^3^, respectively, while they were 37.10 μg/m^3^, 15.20 μg/m^3^, 22.90 μg/m^3^ and 9.50 μg/m^3^ in the hot season. What’s more, the trend of total respiratory outpatient visits in different seasons were similar to the daily air pollutant concentrations.

### Associations between air pollutants and outpatient visits for respiratory diseases

**Fig. 3:**
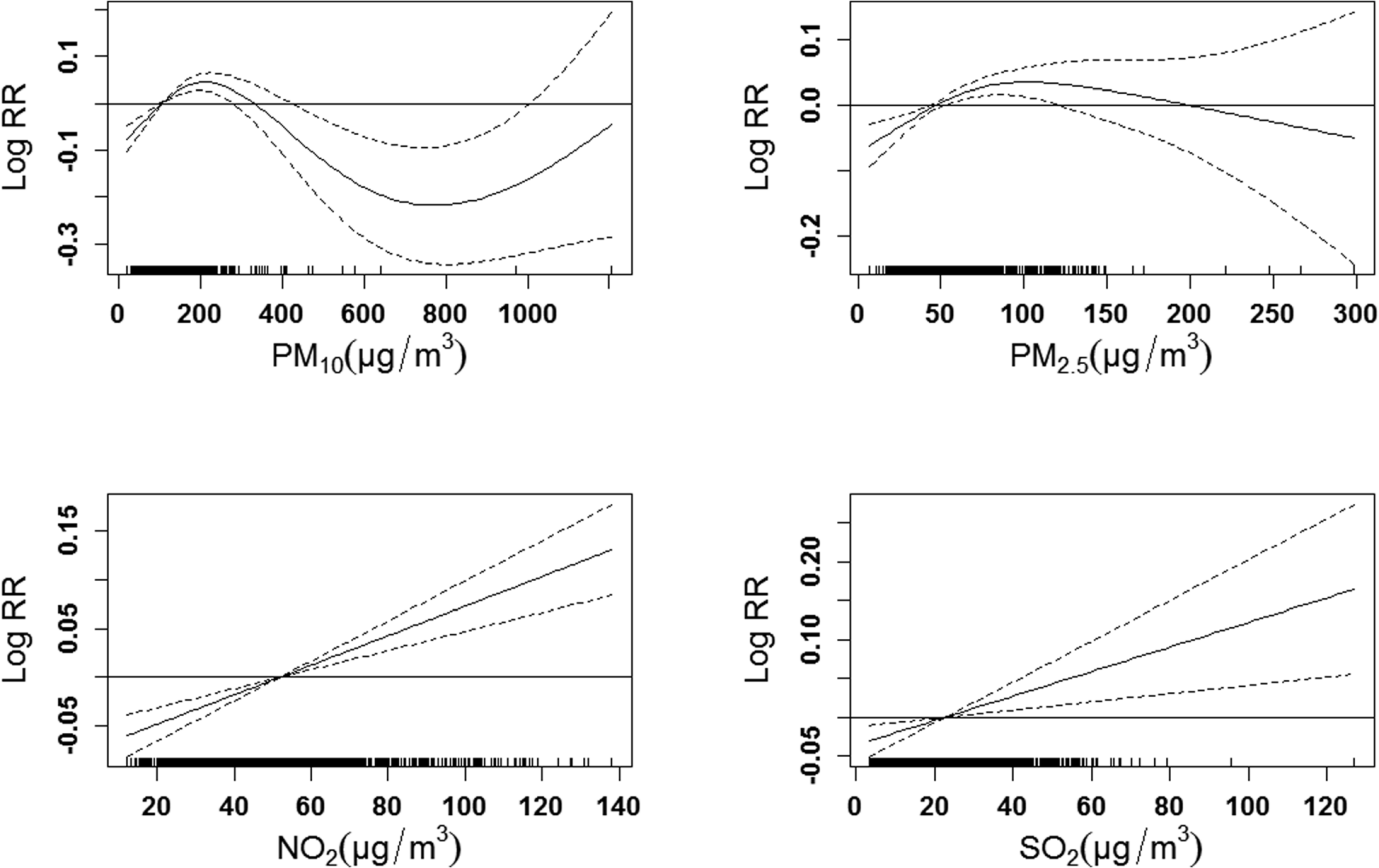
The exposure-response curves of air pollutants concentrations and total outpatients with respiratory diseases in Lanzhou, China, during 2014–2016. The X-axis is the concurrent day air pollutants concentrations (μg/m^3^), Y-axis is the predicted log relative risk (RR), is shown by the solid line, and the dotted lines represent the 95% confidence interval (CI). PM_2.5_, particulate matter with aerodynamic diameter ≤ 2.5 μm; PM_10_, particulate matter with aerodynamic diameter ≤ 10 μm; NO_2_, nitrogen dioxide; SO_2_, sulfur dioxide

**Fig. 4:**
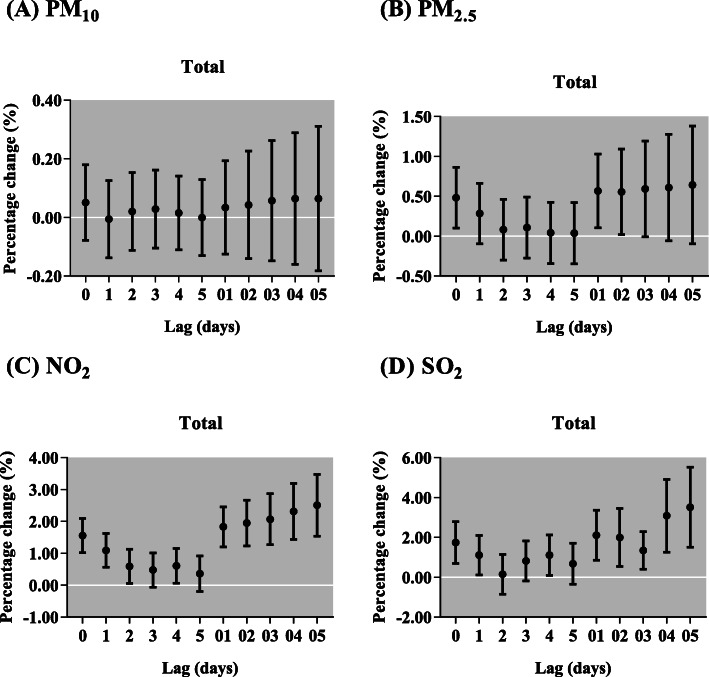
Percentage change (95% confidence interval) of children outpatient visits for total respiratory diseases per 10 μg/m^3^ increase in concentrations of air pollutants for different lag days in the single-pollutant models in Lanzhou, China, during 2014–2016. PM_2.5_, particulate matter with aerodynamic diameter ≤ 2.5 μm; PM_10_, particulate matter with aerodynamic diameter ≤ 10 μm; NO_2_, nitrogen dioxide; SO_2_, sulfur dioxide

**Fig. 5:**
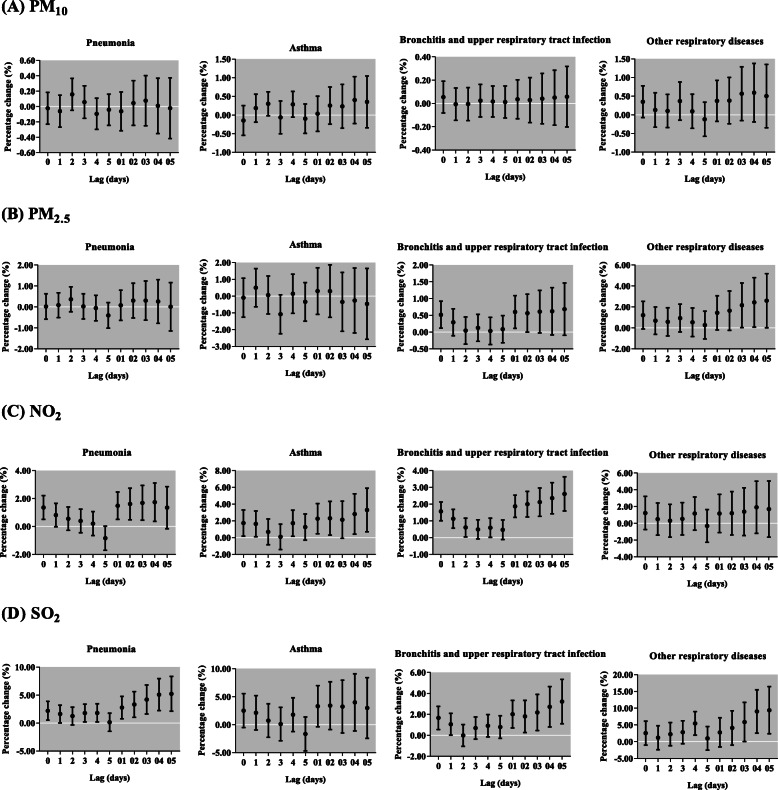
Percentage change (95% confidence interval) of children outpatient visits for cause-specific respiratory diseases per 10 μg/m^3^ increase in concentrations of air pollutants for different lag days in the single-pollutant models in Lanzhou, China, during 2014–2016. PM_2.5_, particulate matter with aerodynamic diameter ≤ 2.5 μm; PM_10_, particulate matter with aerodynamic diameter ≤ 10 μm; NO_2_, nitrogen dioxide; SO_2_, sulfur dioxide

In Fig. 3, we observed significantly positive associations between respiratory diseases outpatient visits and the concentration of NO_2_ and SO_2_. In single-pollutant models, we found PM_2.5_, NO_2_ and SO_2_ were significantly associated with the increased respiratory outpatient visits (Fig. 4). Each 10 μg/m^3^ increase of PM_2.5_ was only significantly associated with total respiratory outpatient visits in lag 0, lag 01 and lag 02. The increments of respiratory outpatient visits were the highest in lag 05 for NO_2_ and SO_2_. The respiratory outpatient visits in lag 05 increased by 2.50% (95% CI: 1.54, 3.48%) and 3.50% (95% CI: 1.51, 5.53%) with per 10 μg/m^3^ increase in NO_2_ and SO_2_, respectively. In cause-specific analysis, PM_2.5_ showed significant effects on the increase of respiratory outpatient visits due to bronchitis and upper respiratory tract infection, and other respiratory diseases, but the significant effect of PM_10_ was not observed in any type of respiratory diseases (Fig. 5). To NO_2_, the significantly positive associations were attributed to pneumonia, asthma, and bronchitis and upper respiratory tract infection, with the greatest increase [1.73% (95% CI: 0.37, 3.11%) in lag 04, 3.28% (95% CI: 0.71, 5.91%) and 2.60% (95% CI: 1.59, 3.63%) in lag 05] in their outpatient visits, respectively. Moreover, for SO_2_, we found the significantly positive associations in pneumonia, bronchitis and upper respiratory tract infection, and other respiratory diseases in lag 05.

**Fig. 6:**
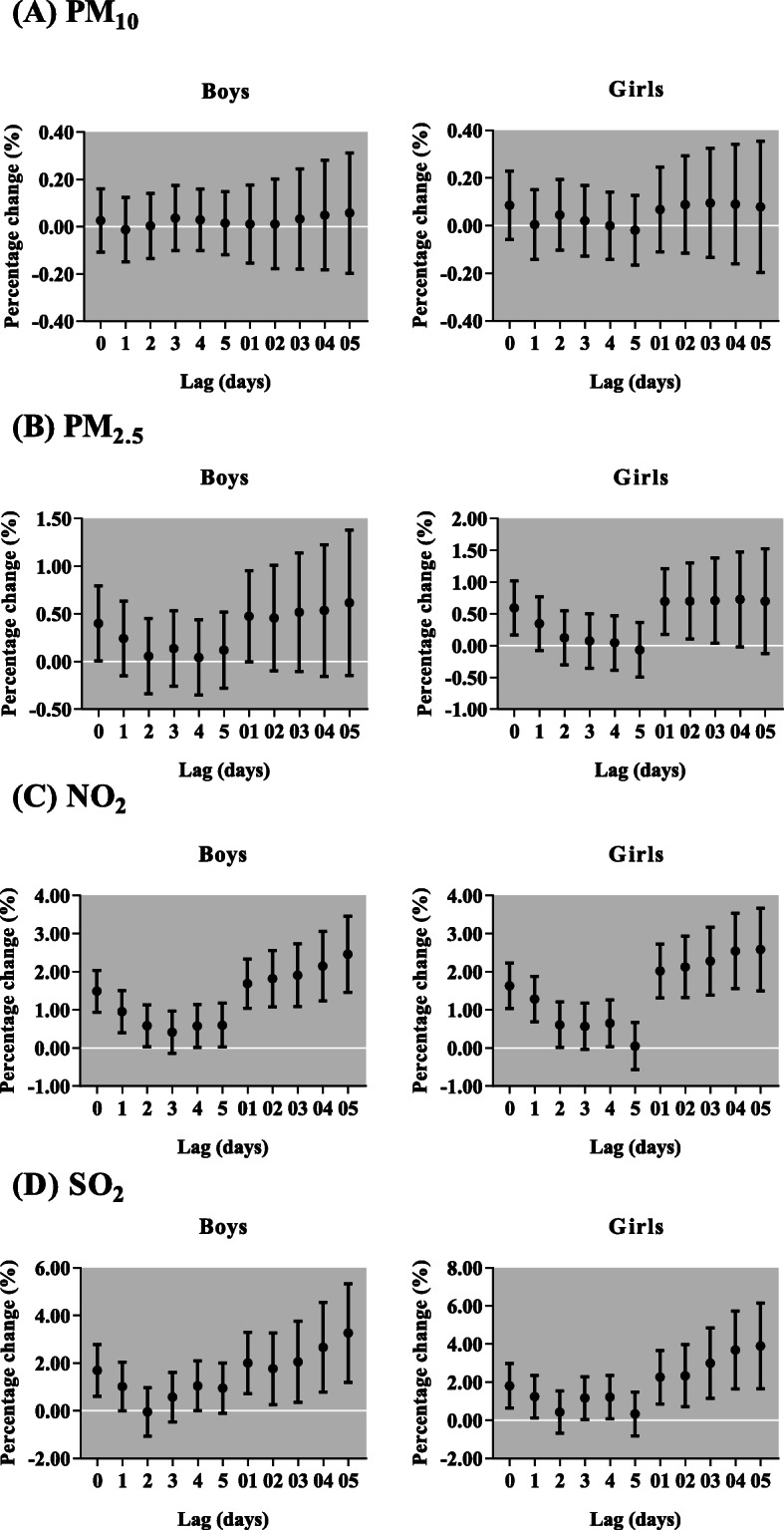
Percentage change (95% confidence interval) of daily children outpatient visits caused by respiratory diseases per 10 μg/m^3^ increase in concentrations of air pollutants stratified by sex for different lag days in the single-pollutant models in Lanzhou, China, during 2014–2016. PM_2.5_, particulate matter with aerodynamic diameter ≤ 2.5 μm; PM_10_, particulate matter with aerodynamic diameter ≤ 10 μm; NO_2_, nitrogen dioxide; SO_2_, sulfur dioxide

**Fig. 7:**
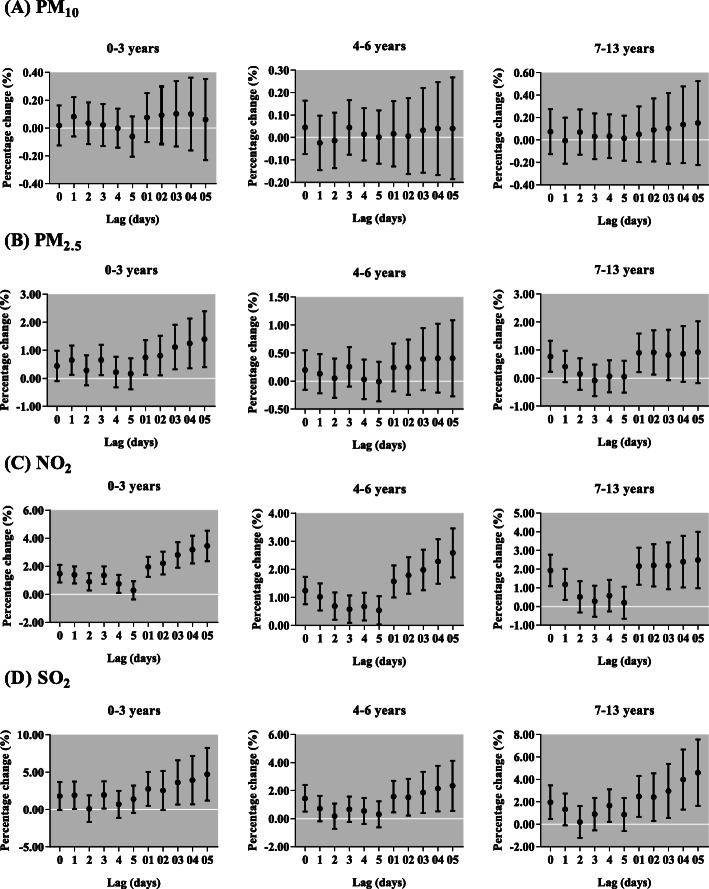
Percentage change (95% confidence interval) of daily children outpatient visits caused by respiratory diseases per 10 μg/m^3^ increase in concentrations of air pollutants stratified by age for different lag days in the single-pollutant models in Lanzhou, China, during 2014–2016. PM_2.5_, particulate matter with aerodynamic diameter ≤ 2.5 μm; PM_10_, particulate matter with aerodynamic diameter ≤ 10 μm; NO_2_, nitrogen dioxide; SO_2_, sulfur dioxide

**Fig. 8:**
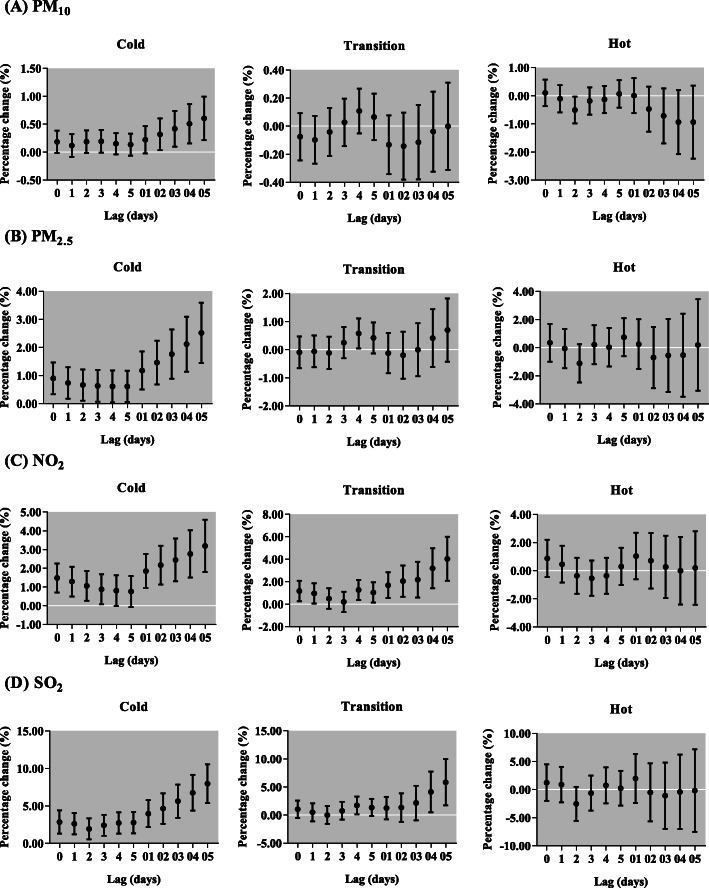
Percentage change (95% confidence interval) of daily children outpatient visits caused by respiratory diseases per 10 μg/m^3^ increase in concentrations of air pollutants stratified by season for different lag days in the single-pollutant models in Lanzhou, China, during 2014–2016. PM_2.5_, particulate matter with aerodynamic diameter ≤ 2.5 μm; PM_10_, particulate matter with aerodynamic diameter ≤ 10 μm; NO_2_, nitrogen dioxide; SO_2_, sulfur dioxide

After sex stratification, we found the effects of PM_10_ on respiratory outpatient visits for both boys and girls were not statistically significant (Fig. 6). However, the increase of each 10 μg/m^3^ in PM_2.5_ was only significantly associated with respiratory outpatient visits for boys in lag 0, but for girls in lag 0, lag 01, lag 02 and lag 03. Each 10-μg/m^3^ increment of NO_2_ and SO_2_ was positively associated with respiratory outpatient visits for boys, with the greatest increase in lag 05 [2.46% (95% CI: 1.46, 3.46%) and 3.25% (95% CI: 1.20, 5.34%), respectively, and girls, with the greatest increase in lag 05 [2.58% (95% CI: 1.50, 3.67%) and 3.89% (95% CI: 1.66, 6.16%), respectively. In different ages, NO_2_ and SO_2_ were positively related to respiratory outpatient visits for all ages, but PM_2.5_ only in children aged 0–3 and 7–13 years (Fig. 7). The effect of NO_2_ was the highest among 0–3 years children in lag 05 [3.45% (95% CI: 2.37, 4.54%)]. Meanwhile, the maximum increase of respiratory outpatient visits due to a 10 μg/m^3^ increase of SO_2_ occurred in lag 05 in children aged 0–3 [4.67% (95% CI:1.22, 8.24%)]. In addition, the greatest increment of respiratory outpatient visits was occurred in lag 05 with a 10 μg/m^3^ increase of PM_10_ [0.60% (95% CI: 0.21, 0.99%)], PM_2.5_ [2.52% (95% CI: 1.45, 3.60%)] and SO_2_ [7.95% (95% CI: 5.40, 10.55%)] in the cold season, but NO_2_ [4.02% (95% CI: 2.08, 5.99%)] in the transition season (Fig. 8). The positive associations were observed among air pollutants, including PM_2.5_ with PM_10_ (*r* = 0.73), SO_2_ (*r* = 0.60) and NO_2_ (*r* = 0.57); and PM_10_ with SO_2_ (*r* = 0.33) and NO_2_ (*r* = 0.39); SO_2_ with NO_2_ (*r* = 0.53) (Table 3).

**Table 3:** Pearson correlation analysis of pollutants

Pollutants	PM_2.5_	PM_10_	SO_2_	NO_2_
PM_2.5_	1.00			
PM_10_	0.73^*^	1.00		
SO_2_	0.60^*^	0.33^*^	1.00	
NO_2_	0.57^*^	0.39^*^	0.53^*^	1.00

^*^P < 0.05

After the optimum lag day for each pollutant being determined in the single-pollutant models, the two-pollutant models were used to adjust for other pollutants. Table 4 compared the results of the single-pollutant models with the results of the two-pollutant models using exposure in lag 05 after adjusting for other pollutants. After adjusting for PM_10_ and PM_2.5_ concentration in the two-pollutant models, the percentage increase for total respiratory diseases outpatient visits of NO_2_ and SO_2_ remained statistically significant with a little increase. However, after controlling NO_2_ and SO_2_, we found the percentage changes of PM_2.5_ and PM_10_ were not statistically associated with total respiratory diseases outpatient visits, similar to the results of the single-pollutant models.

**Table 4:** Percentage change (95% confidence interval) of total children respiratory outpatients per 10 μg/m^3^ increase in concentrations of pollutants in the single and two-pollutant models

Pollutants	Single- and Two-pollutant models	Estimates
PM_10_	PM_10_	0.06 (−0.18, 0.31)
	PM_10_ + NO_2_	−0.12 (− 0.38, 0.13)
	PM_10_ + SO_2_	−0.04 (− 0.29, 0.22)
PM_2.5_	PM_2.5_	0.64 (− 0.10, 1.38)
	PM_2.5_ + NO_2_	−0.47 (−1.32, 0.38)
	PM_2.5_ + SO_2_	−0.08 (− 0.94, 0.79)
NO_2_	NO_2_	**2.50 (1.54, 3.48)**
	NO_2_ + PM_10_	**2.64 (1.63, 3.65)**
	NO_2_ + PM_2.5_	**2.83 (1.70, 3.97)**
	NO_2_ + SO_2_	**2.17 (1.10, 3.26)**
SO_2_	SO_2_	**3.50 (1.51, 5.53)**
	SO_2_ + PM_10_	**3.57 (1.52, 5.65)**
	SO_2_ + PM_2.5_	**3.62 (1.26, 6.03)**
	SO_2_ + NO_2_	1.49 (−0.68, 3.70)

*PM*_*2.5*_ particulate matter with aerodynamic diameter ≤ 2.5 μm, *PM*_*10*_ particulate matter with aerodynamic diameter ≤ 10 μm, *NO*_*2*_ nitrogen dioxide, *SO*_*2*_ sulfur dioxide. Six-day moving average (lag 05) concentrations of pollutants were used. Bold values denote significant differences

## Discussion

Lanzhou has a population of over 3.7 million with children accounting for 14% in 2016 [32]. In this study, we observed 332,337 children’s outpatient visits for respiratory diseases within 3 years, suggesting respiratory diseases is a major health problem among children in Lanzhou. Many studies about air pollution and children respiratory diseases were conducted in the cities of China with moist climate, such as Shenzhen [33], Hefei [34] and so on. However, research about comprehensive comparison of air pollution at respiratory diseases of different groups (gender, age, season and cause-specific diseases) is still limited, especially in city with arid climate. Therefore, our results may add to the limited scientific knowledge that air pollution may also affect the incidence of respiratory diseases among children from different subgroups in an arid climate city.

The results showed that PM_2.5_, NO_2_ and SO_2_ were significantly associated with the increased total respiratory outpatient visits of children. A study in Shanghai during 2013–2015 found that an interquartile range (IQR) increase in PM_2.5_, SO_2_ and NO_2_ was associated with a 8.81, 17.26 and 17.02% increase for daily pediatric respiratory emergency visits in lag 03, respectively [35], which is higher than our study. The possible explanation is that the air pollution level in Shanghai of 2013–2015 showed a trend of rising, but it has been persistently declining in Lanzhou since 2013 [36]. However, a study conducted in Yichang during 2014–2015, China, observed that each IQR increase in PM_2.5_ and NO_2_ concentrations corresponded to a 1.91 and 1.88% increase of pediatric respiratory outpatient visits at current day, respectively [37], which was higher for PM_2.5_ but lower than our study for NO_2_. It is because that the daily average concentration of PM_2.5_ in Yichang was higher (84.9 μg/m^3^ VS 54.52 μg/m^3^) but NO_2_ was lower than Lanzhou (37.4 μg/m^3^ VS 51.80 μg/m^3^) [37]. However, the associations between PM_10_ and total respiratory outpatients were insignificant, which is not consistent with the findings from other studies [35, 37]. Shanghai is characterized by a higher degree of urbanization and industrialization than Lanzhou, so the PM_10_ of which mainly comes from traffic and industry pollution sources, similar to that in Yichang [36, 38]. However, the PM_10_ in Lanzhou was mainly contributed by raised dust containing higher level of crustal elements, which is not as poisonous as that in Shanghai and Yichang [39]. Even so, our results indicate that the air pollution is positively related to the respiratory diseases among children in Lanzhou.

It is well known that air pollutants are the risk factors for many respiratory diseases in children. An eight-year time-series study in Hanoi showed that all air pollutants (PM_10_, PM_2.5_, NO_2_ and SO_2_) were positively associated with pneumonia, bronchitis and asthma hospitalizations among children [18], like that reported in Shijiazhuang [23] and Taiwan [9]. Consistent with these studies, we also found all air pollutants (except PM_10_) were positively related to the outpatient visits of bronchitis and upper respiratory tract infection. Coupled with the fact that bronchitis and upper respiratory tract infection were the major types of respiratory diseases (87.45% of all respiratory outpatient visits) in Lanzhou, the effect of air pollution may explain part of this phenomenon. To asthma, gas pollutant like NO_2_ has been well known as its major risk factor, which has also been confirmed in a study with a broad range of exposures and diverse populations among children published in the Lancet [40]. Unfortunately, similar result was also found in our study with an arid climate. Therefore, although the Lanzhou government has worked positively and gained great international compliment in reducing the air pollution [41], more efforts will be needed to reduce the air pollution from vehicle exhaust.

In the stratified analysis, the impact of air pollution was more significant on girls than boys, which is consistent with the study in Taiwan among the children respiratory outpatients [9]. A review showed that girls had smaller lungs, shorter and wider airways, and exhibited higher forced expiratory flow rates than boys [42]. Therefore, the airways of girls may be less able to block air pollution. However, there is lack of consistent results for sex differences in health effects of various air pollutants. Many similar studies conducted in Beijing for asthma children [43], in Ningbo for respiratory infections children [44], in Jinan for outpatient respiratory diseases [45] and in Hanoi for children lower respiratory infections [18] found that there was no obvious difference between boys and girls. Thus, additional studies are needed to clarify whether there are sex differences for the associations between air pollutants and respiratory diseases among children. In age difference, we found younger children (0–3 years) were more vulnerable to air pollution. A study in Ningbo for pneumonia observed stronger associations between air pollutants and children under 5 years [17]. The study in Hanoi also showed positive relationship between airborne particles and daily hospital admission for respiratory diseases among children aged < 5 years [46]. It is generally recognized that this high vulnerability among younger children can be attributed to their immature lungs, higher breathing rate [47] and predominantly oral breathing characteristics [48], which increased their exposure and susceptibility to respiratory infections. These factors, combined with the underdeveloped immune function, may add together to make infant and younger children more susceptible to air pollutants.

In the present study, the descriptive results showed that the concentrations of air pollutants in Lanzhou were higher in the cold season, which is consistent with the study in Shennongjia [49]. Previous study suggested winter was the most polluted season [50]. In Northeastern and Northwestern China, due to a specific cold climate in winter and regional living habits, air pollution mainly comes from coal burning, motor vehicles and industrial production [51, 52]. Lanzhou is located in Northwestern China with a narrow and long valley basin and low winds, stable stratification especially inversion, which blocks the air streams and makes the pollutants difficult to disperse [53]. In addition, coal use in the winter also increases the level of air pollution [36]. These factors may lead the air pollution of Lanzhou to be the most severe in cold seasons. This may explain why we found the greatest effects of PM_10_, PM_2.5_ and SO_2_ on children respiratory outpatients in the cold season. However, the result for NO_2_ agrees with the similar study in Shijiazhuang [23], but is inconsistent with the study in Yichang [37]. This may also be explained by the different source of air pollutants among these cities, NO_2_ was also the major air pollutant in Yichang but not in Lanzhou or Shijiazhuang.

Our study has several limitations. First, the study only used the data of 3 years due to limited data accessibility and availability, which may not be abundantly enough to evaluate the effects of air pollution on child respiratory outpatients, but we at least provide some hypothesis from a specific topography with an arid climate and large sample. Second, the air pollution data were collected from only four monitoring stations, the average value of which may not be strong enough to represent the real condition of air quality in Lanzhou. Thus, there should be data from more monitoring stations of Lanzhou. Third, unknown or unmeasured confounders such as indoor air pollution, second-hand smoke exposure and so on may exist and affect the associations. Therefore, all these limitations should be solved in future studies.

## Conclusions

Our results indicate that the air pollution exposure may account for the increased risk of outpatient visits for respiratory diseases among children in Lanzhou, particularly for younger children and in the cold season. To our knowledge, this is the first study to investigate the short-term effects of air pollution on child respiratory morbidity based on the large population in Northwestern China. The estimated percent changes may be helpful to monitor the disease burden caused by air pollution in Lanzhou among children and strengthen the urgency for controlling air pollution in Lanzhou. Since children are much more susceptible to air pollution, more urgent strategies will be needed to deal with the higher level of respiratory diseases among children, like promoting the use of personal protective equipment (e.g., respirators, air purifiers) and avoiding outdoor activities during heavily polluted weathers of Lanzhou.

## Data Availability

The datasets used and/or analyzed during the current study are not publicly available but are available from the corresponding author on reasonable request.

## Abbreviations

PM_10_: Particulate matter with aerodynamic diameter ≤ 10 μm
PM_2.5_: Particulate matter with aerodynamic diameter ≤ 2.5 μm
NO_2_: Nitrogen dioxide
SO_2_: Sulfur dioxide
GAM: Generalized additive model
CI: Confidence interval
RR: Relative risk
